# The efficacy of an irrigating eyelid retractor-facilitated ocular rinse on MMP-9 expression and dry eye disease

**DOI:** 10.1016/j.aopr.2024.05.002

**Published:** 2024-05-20

**Authors:** Natasha Mayer, Srinivas Sai A. Kondapalli, Nandini Venkateswaran, Hajirah N. Saeed

**Affiliations:** University of Pittsburgh School of Medicine, Pittsburgh, PA, USA; Everett and Hurite Ophthalmic Association, Pittsburgh, PA, USA; Department of Ophthalmology, Massachusetts Eye and Ear, Boston, MA, USA; Department of Ophthalmology, University of Illinois of Chicago, Chicago, IL, USA

Dear Editor,

Dry eye disease (DED) is a chronic inflammatory condition of the ocular surface that can result in reduced vision and irritation. Dysregulation of the ocular surface can occur from a variety of sources, including, but not limited to aging, inflammatory diseases, surgeries, and medications, all of which can decrease tear secretion and increase inflammation on the ocular surface and/or meibomian glands. This reduction in tear secretion can lead to a hyperosmolar tear which is a hallmark of chronic DED.^1^ This hyperosmolar tear state initiates an inflammatory response on the ocular surface. The inflammatory response results in the release of inflammatory cytokines, including interleukin (IL)-1, IL-6, and tumor necrosis factor-a.^2^ These cytokines trigger the upregulation of matrix-metalloproteinase-9 (MMP-9), which consequently affects the corneal barrier epithelium.^3^ MMP-9 elevation in the tear lake, therefore, is an indicator of inflammation on the ocular surface.

Point-of-care (POC)^1^ immunoassays for MMP-9 (InflammaDry®, QuidelOrtho San Diego, CA, USA) were introduced in 2013. This semi-quantitative test evaluates the tear lake via sterile sampling of the inferior conjunctival forniceal tear lake. After interaction with a reagent, the test indicates a positive result in the presence of a threshold level of at least 40 ng/mL of MMP-9.^4^

Clinically, POC MMP-9 testing is widely used as an in-office tool to evaluate elevated ocular surface inflammation, which is suggestive of DED. Irrigation of the ocular surface has been investigated by others and has been shown to reduce MMP-9 and improve the symptoms of DED.^5^, ^6^, ^7^, ^8^ Diaz et al. investigated eye irrigation in a randomized controlled trial compared to standard artificial tears.^7^ The trial noted a 68% reduction in dry eye symptoms, based on Ocular Surface Disease Index (OSDI) scores. Other groups have illustrated that daily eye rinsing can be beneficial to reduce dry eye symptoms as illustrated with improved OSDI and dry eye questionnaire 5 in addition to reduction in MMP-9 levels.^5^^,^^6^ However, in studies investigating eye irrigation, irrigations were repeated, patient-directed, and required at least 21 days duration of continuous therapy.^5^, ^6^, ^7^, ^8^

An irrigating eyelid retractor is a novel medical device that aims fluid towards the palpebral conjunctiva, conjunctival fornix, and bulbar conjunctiva. This device is the first of its kind which aims fluid specifically at the palpebral conjunctiva and fornix, areas that are anatomically difficult to reach using standard irrigation. The device is a single use, sterile medical device made of acrylonitrile butadiene styrene medical grade plastic and has been shown to provide a superior rinse compared to standard manual irrigation in patients post-intravitreal injections.^9^ The device is inserted underneath the eyelid and jets fluid in three directions to rinse the palpebral conjunctiva, bulbar conjunctiva, and conjunctival fornix. The device does not require patient participation once inserted underneath the eyelid. Standard irrigation practices can be limited due to patient anatomy (a prominent eyebrow, deep set orbit, small tight eyelids) as well as patient cooperation (a patient who squeezes their eyelids shut). Therefore, the hypothesis is with the irrigating eyelid retractor there should be a greater reduction in MMP-9 compared to a standard rinse. Our purpose was to 1) investigate the effect of a single ocular rinse with an irrigating eyelid retractor on MMP-9 levels at 3 hours post-rinse compared to the standard rinse protocol, 2) evaluate the effect of the eyelid irrigation device on clinical symptoms of DED using the (Change in Dry Eye Severity Questionnaire (CDES-Q) at 1-week post-rinse. 3) determine the potential long-term benefits of a device-assisted ocular rinse.

## 1 Methods

The study was approved by Advarra IRB (Columbia, Maryland USA) in accordance with the tenets of the Declaration of Helsinki and the Health insurance Portability and Accountability Act (HIPAA) of 1996. Informed consent was obtained with IRB oversight. In This multicenter, randomized prospective study, patients ≥18 years of age with symptoms of DED and a positive POC MMP-9 test in at least one eye were enrolled at their regularly scheduled visit. Subjects were then randomized to either device-assisted irrigation arm of standard irrigation (control arm). Dry eye symptoms were characterized by the presence of any burning, irritation, redness, sandiness, and/or light sensitivity.^1^^,^^10^ Patients on anti-inflammatory medication, those who reported using artificial tears or other topical ocular medications within 14 days prior to enrollment, and those who began new topical or oral anti-inflammatory medication after enrollment were excluded. Patients were also excluded if they had undergone intraocular surgery within the past 6 months, worn contact lens within the past 12 hours, had a history of acute allergic or infectious conjunctivitis, current punctal plug use, Stevens-Johnsons syndrome, cicatricial conjunctival disease, or severe dry eye preventing wetting of the POC testing as per guidelines of the MMP-9 POC test recommendations. Pregnant or lactating patients were excluded from the study. Previous cataract or refractive surgery more than 6 months prior to enrollment was not exclusionary.

**Fig. 1:**
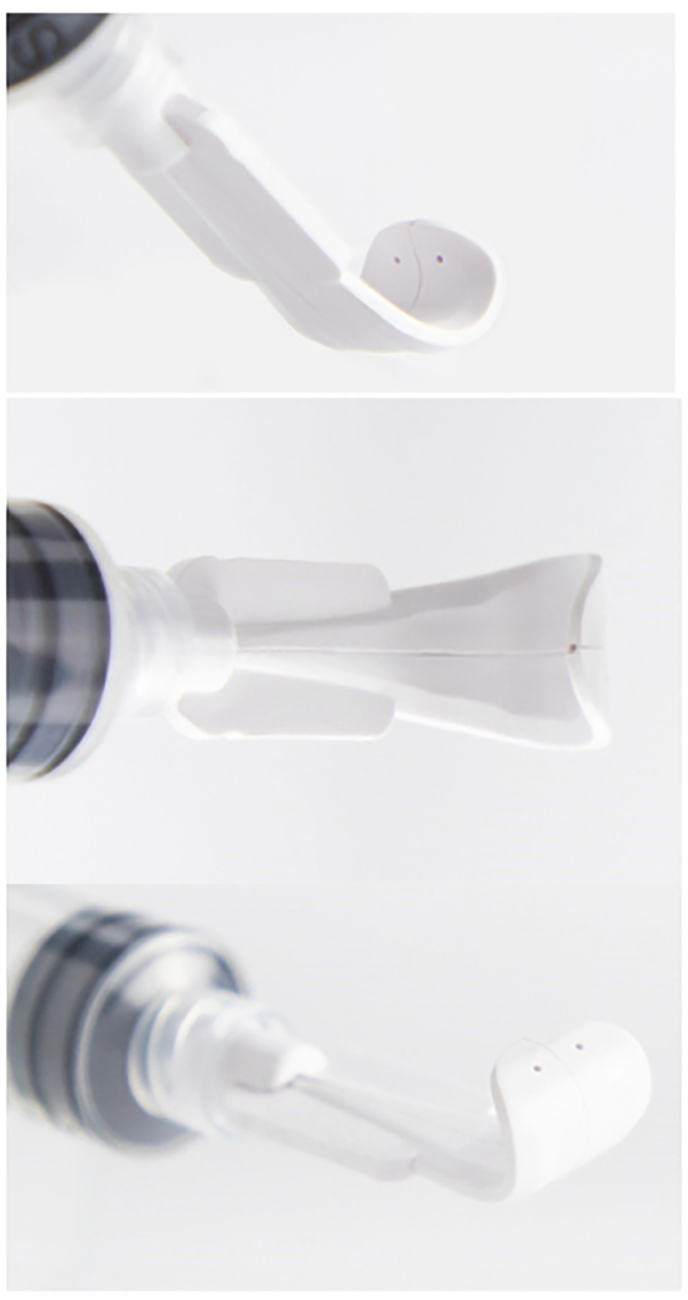
Irrigating eyelid retractor illustrating ports as the distal end aimed at the palpebral conjunctiva, conjunctival fornix and bulbar conjunctiva (top, middle, bottom, respectively).

**Fig. 2:**
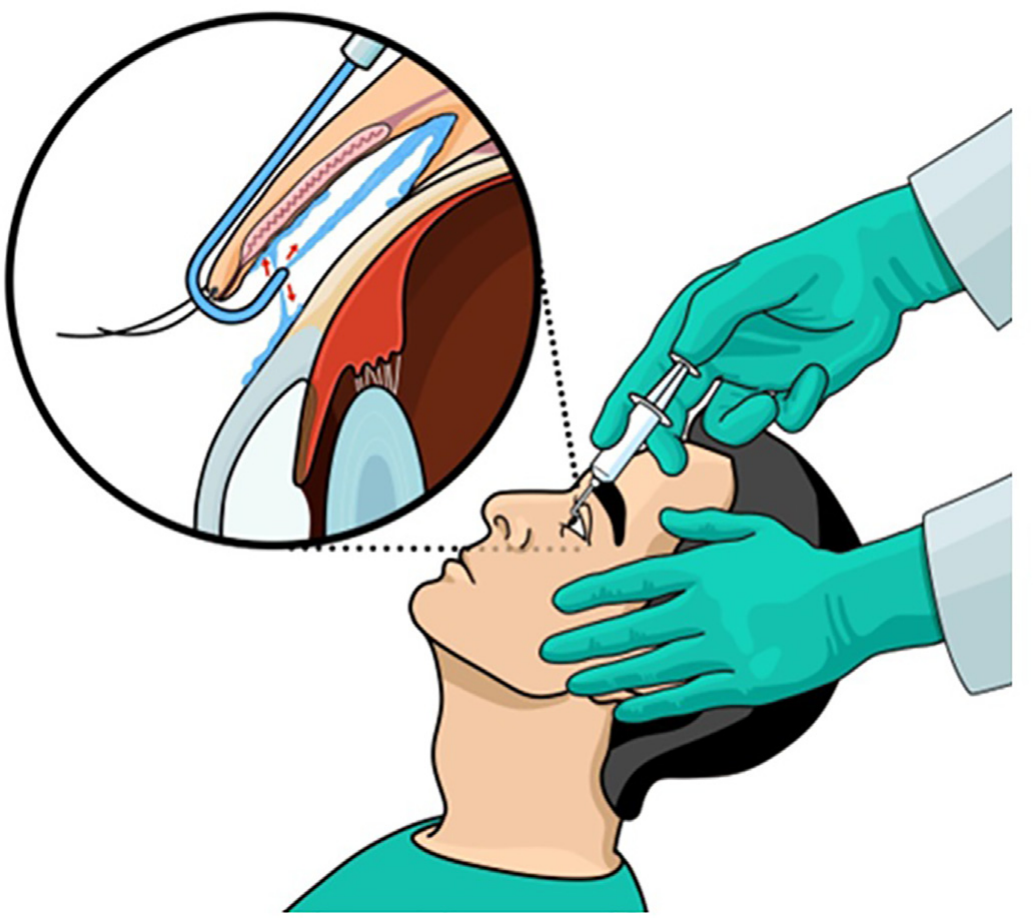
Illustration of the irrigating eyelid retractor under the upper eyelid with arrows (red) demonstrating the direction of fluid aimed simultaneously at the palpebral conjunctiva, conjunctival fornix and bulbar conjunctiva.

Eye irrigation was performed via irrigating eyelid retractor or manual eyelid retraction (control arm) on the same day as the initial MMP-9 testing. For randomization an online randomization tool was employed which grouped patients into a standard rinse or device rinse (www.randomizer.org). For both procedures, 15mL of eyewash solution (McKesson® Richmond, VA, USA) of 98.3% purified water was used as a rinse medium. This eyewash solution contained inactive ingredients of boric acid, sodium borate, and sodium chloride. The irrigating eyelid retractor is a sterile, single-use medical device composed of biocompatible acrylonitrile butadiene styrene plastic. The proximal end of the device has a luer-lock that fits onto a syringe filled with eye wash solution. Once the plunger of the syringe is depressed, fluid exits via the five ports at the distal end of the irrigating retractor. The device has two ports directed towards the palpebral conjunctiva, two ports directed toward the bulbar conjunctiva and one port toward the conjunctival fornix (Fig. 1). Therefore, the fluid is specifically aimed perpendicular to the palpebral conjunctiva and conjunctival fornix (Fig. 2).

For patients in the device-assisted irrigation arm, the upper eyelid was gently manually retracted upward, while the patient was instructed to look down. The retractor was then placed underneath the upper eyelid. The plunger of the syringe was depressed, and the retractor was moved horizontally along the lid margin; 10mL of eyewash solution was used. The person administering the rinse was instructed to push the 10mL of fluid in 10 seconds as measured by a digital stopwatch. Subsequently, the device was removed from the upper eyelid. The same procedure was performed for the lower eyelid. Again, the lower eyelid was manually retracted downward, the patient was instructed to look up. Care was used in placement of the device underneath the lower eyelid. The plunger of the syringe was depressed, and 5mL of eyewash solution was used. The person administering the rinse was instructed to push the 5mL of fluid in 5 seconds as measured by a digital stopwatch. When the plunger was depressed, again the device was moved horizontally along the eyelid margin. The device and syringe were then disposed of in biohazard waste.

For patients in the manual irrigation arm, a syringe filled with eye wash solution was directed towards the bulbar conjunctiva. The upper eyelid was retracted with the patient looking down, and irrigation was performed using 10mL eyewash solution. The person administering the rinse was instructed to push the 10mL of fluid in 10 seconds as measured by a digital stopwatch. The lower eyelid was then retracted and irrigation was performed using 5mL of eyewash solution. The person administering the rinse was instructed to push the 5mL of fluid in 5 seconds as measured by a digital stopwatch. We decided to use 10mL of eyewash solution in the upper eyelid as it covers 67% of the cornea and globe while the lower eyelid covers 33% of the cornea and globe. Certified ophthalmic assistants and board-certified ophthalmologists were trained to administer the rinses.

Three hours post-irrigation repeat testing was performed. Three hours was determined the appropriate time after rinse given that the MMP-9 POC test requires at least 2 hours after administration of any medication for the test to be reliable.^11^ Between irrigation and initial repeat testing, the patients were kept in the same office space and instructed not to use any eye drops. As an exploratory endpoint, in the device group, repeat testing was also performed 7 days post-irrigation as well as 30–91 days post-rinse, depending on patient availability.

Patients were given the Change in Dry eye Severity Questionnaire (CDES-Q) on day 7 post-irrigation. The CDES-Q is a validated questionnaire to evaluate changes in dry eye symptoms.^16^ The CDES-Q has two questions. Question 1 asked the subject whether they felt better, worse, or the same compared to their initial visit. If they indicated a better or worse status, question two asked the subject to score their improvement/worsening on a scale from 0 to 10, where 0 meant slightly better or worse, and 10 meant extremely better or worse.

**Fig. 3:**
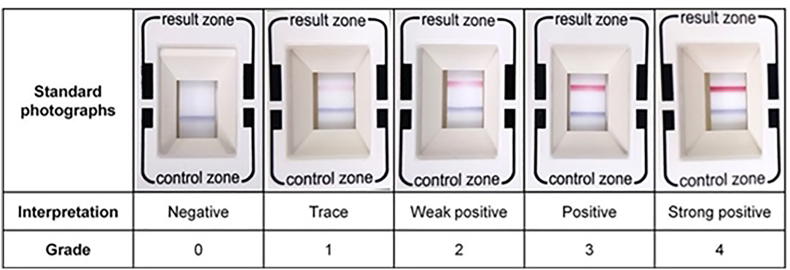
*Digital photograph of ImflammaDry MMP-9 immunoassay.* Blue lines indicate control condition and red lines indicate MMP-9 detection. Red line intensity indicates relative amount of MMP-9 detected with more intense red lines revealing higher MMP-9 expression.

Digital photographs of the MMP-9 tests were obtained, de-identified, randomized, and sent to four blinded graders. Graders were given a standardized grading scale developed by Kim et al.^12^ The scale ranges from 0 to 4, with 0 indicating ≤40 ng/mL MMP-9 (lack of a red band) and 4 indicating ≥5000 ng/mL MMP-9 (dark red band) (Fig. 3). All 4 graders were clinically practicing ophthalmologists or optometrists.

A pilot study of 20 participants was conducted prior to data collectionto ascertain the parameters required for a subsequent power calculation. 20 was predetermined to be the number for the pilot study based on the statistical literature.^13^ This preliminary investigation served to evaluate the feasibility and effectiveness of the research design, methodology, and data collection procedures. A preliminary mean and standard deviation to run a power analysis was calculated through an analysis of the pilot study results. These findings were instrumental in determining the appropriate sample size necessary to achieve the desired statistical power for detecting meaningful effects in the subsequent larger-scale study.

## 2 Results

### 2.1 Group characteristics

Eighty-eight total eyes of 48 participants were enrolled; 26 participants in the device arm and 22 in the standard rinse arm. Enrollment began in April 2022 in five sites, in the states of Ohio, West Virginia and Pennsylvania. Enrollment ended in February 2023. Table 1 illustrates the demographic data of participants. Forty-six eyes (52.27%) underwent device-facilitated irrigation and 42 eyes (46.73%) underwent standard irrigation. Five eyes (10.87%) in the device group and 2 eyes (4.76%) in the standard group were lost to follow-up 3 hours post-irrigation. No difference was found between baseline MMP-9 expression and rankings, as reported by independent graders (*P* ​> ​0.05).

**Table 1:** Demographics of participants.

	Device Rinse	Standard Rinse
Total Participants	26	22
Women, n (%)	23 (88.41%)	19 (86.36%)
Race, n (%)	Caucasian, 20 (76.92%)	Caucasian, 19 (86.36%)
	Black, 1 (3.85)	Black, 3 (13.63%)
	Asian, 5 (19.23%)	Asian, 0 (0%)
Age	43.23 ​± ​3.42	42.00 ​± ​3.02

### 2.2 Three hours post-rinse

**Fig. 4:**
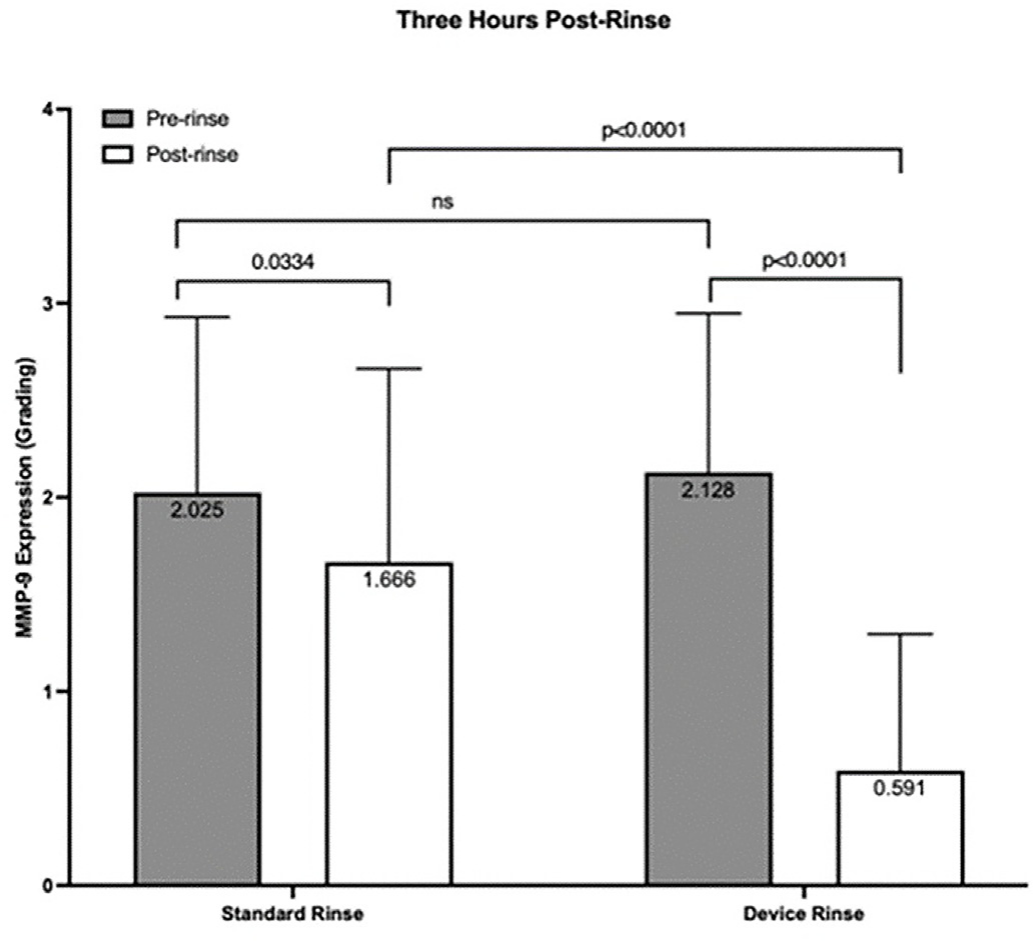
*MMP-9 grading as compared to baseline for control and device rinse groups for 3 hours post-rinse*. Grey bars indicate baseline MMP-9 expression reported as the average grade of 4 independent graders. No significant difference (ns) was observed between baseline groups (*P* ​> ​0.05). Error bars indicate standard deviation and columns are labeled with the average graded value for each group. Both control and device groups showed a reduction in MMP-9 expression (*P* ​= ​0.0334 and *P* ​< ​0.0001, respectively) compared to baseline. Combination effects of time and protocol reveal significant reduction in MMP-9 post-rinse in the device group as compared to the control condition (*P* ​< ​0.0001).

Table 2 illustrates the mean values for eyes enrolled in the study before and after device or standard rinse, respectively. The mean at baseline showed no significant difference among groups (control arm:2.025 ​± ​0.904, treatment arm:2.128 ​± ​0.820; *P* ​> ​0.05). Both groups showed a reduction in MMP-9 expression post-irrigation, with the treatment arm reaching statistical significance (control arm:1.666 ​± ​0.006, *P* ​= ​0.0334; treatment arm:0.591 ​± ​0.704, *P* ​< ​0.0001, respectively). Two-way repeated-measures ANOVA revealed a statistically significant interaction effect between time (before and after rinse) and group (control and treatment arm), indicating that the device rinse showed a significant reduction in MMP-9 expression post-rinse as compared to the control group (*P* ​< ​0.0001) (Fig. 4). 20 eyes (44%) in the device arm were negative at 3 hours post-rinse compared to 1 eye (2.3%) in the standard irrigation arm. In the device arm, 20 (43.47%) subjects were negative 3 hours post rinse; in the standard rinse 1 (2.38%) subject was negative 3 hours post rinse.

**Table 2:** Mean values on a 0–4 scale of MMP-9 via POC testing before and after device and standard rinse, respectively.

Participant Number	Pre-Device Rinse	Post-Device Rinse	Participant Number	Pre-Standard Rinse	Post-Standard Rinse
1	1.75	0.00	1	2.00	2.33
2	2.25	0.75	2	1.67	1.00
3	2.25	0.00	3	3.00	0.00
4	1.25	0.00	4	1.33	1.67
5	3.00	1.25	5	1.67	2.00
6	3.25	0.50	6	1.00	1.00
7	3.75	1.00	7	2.00	0.33
8	2.25	0.25	8	2.00	0.33
9	2.75	0.00	9	2.00	2.33
10	2.50	0.00	10	3.33	1.33
11	2.00	0.00	11	1.00	0.33
12	2.00	1.75	12	1.00	0.33
13	2.00	1.00	13	1.33	1.33
14	2.50	1.00	14	3.67	2.67
15	1.00	0.00	15	1.00	2.00
16	1.75	1.00	16	0.67	1.00
17	2.25	0.75	17	1.00	1.00
18	3.25	0.00	18	2.00	0.67
19	2.75	0.75	19	2.00	1.00
20	2.25	1.50	20	1.67	0.67
21	1.25	1.00	21	1.00	0.33
22	1.25	0.00	22	2.67	1.33
23	1.00	0.00	23	2.33	2.67
24	3.00	3.00	24	3.33	1.33
25	1.75	1.00	25	3.00	1.67
26	1.00	0.00	26	1.33	1.33
27	2.00	1.00	27	2.00	1.33
28	1.00	0.00	28	1.67	2.00
29	1.25	1.00	29	2.00	3.33
30	2.00	1.00	30	2.33	2.67
31	2.00	0.00	31	4.00	2.33
32	2.25	0.00	32	2.33	2.33
33	2.00	0.00	33	3.00	2.67
34	1.00	0.00	34	2.33	1.67
35	1.00	0.50	35	1.33	1.67
36	2.50	1.00	36	1.67	1.33
37	3.75	0.00	37	3.67	4.00
38	3.50	0.00	38	3.67	4.00
39	3.75	1.00	39	1.00	2.33
40	1.75	0.00	40	1.00	3.00
41	1.50	2.25

### 2.3 Patient symptoms questionnaire

At one week, the average value for the device arm in the CDES-Q was 4.33±2.93. The average value for the standard rinse in the CDES-Q was 1.32±1.21. The difference in means was 1.98e-8 (*P* ​< ​0.05).

### 2.4 Long-term effects of single ocular rinse

Exploratory secondary endpoints in only the device arm to evaluate the durability of MMP-9 reduction were investigated as only a single subject in the standard rinse arm converted to negative 3 ​h post rinse. Check points for long-term MMP-9 reduction were measured at 7 days, and again between 30 and 91 days. Of the 46 eyes in the device arm, 33 (72%) were tested for MMP-9 expression on day 7. Of these 33 eyes, 14 (42%) were negative for MMP-9 expression compared to 44% for 3 hours post-rinse. At 30–91 days post device rinse, the same 33 eyes were tested. Of these, 11 (33%) eyes were negative for MMP-9 expression.

## 3 Discussion

MMP-9 is a hallmark indicator of ocular surface disease inflammation. In our study, we investigated whether a single complete ocular rinse via irrigating eyelid retractor could reduce MMP-9 levels, as measured by POC testing, compared to standard irrigation. At 3 hours post-rinse, there was a statistically significant reduction in MMP-9 by complete ocular irrigation facilitated by an irrigating eyelid retractor compared to standard eye irrigation. The greater reduction of ocular surface inflammation by the device could be due to the standard rinse failure to adequately rinse the palpebral conjunctiva and the conjunctival fornix. Standard ocular rinsing requires the healthcare provider to manually retract the eyelid, have a patient cooperatively keep their eye open and only provides on direction of fluid from the tip of the eye wash bottle/syringe onto the bulbar conjunctiva. If the ocular surface were flat then this would be a reasonable approach to rinse in this manner. However, the ocular surface is three dimensional with an obvious convexity. The palpebral conjunctiva and fornix are also difficult to access. This suggests that the palpebral conjunctiva and conjunctival fornix, which are specifically targeted via the irrigating eyelid retractor, may trap an inflammatory load and house a dysregulated biofilm.^14^, ^15^, ^17^ The palpebral conjunctiva and fornix also house a unique microenvironment that is not subject to the mechanical forces of the blink, unlike the cornea or interpalpebral bulbar conjunctiva.^15^ The novel irrigating eyelid retractor simultaneously has three directions of fluid which are aimed at the bulbar conjunctiva, palpebral conjunctiva and conjunctival fornix. This is accomplished by the device's design. The retractor can be attached to a luer locking syringe filled with fluid. When the plunger of the syringe is pushed, the fluid is released through five ports located at the distal end of the retractor device. Two ports are aimed at the bulbar conjunctiva, two ports are aimed at the palpebral conjunctiva and one port is aimed at the conjunctival fornix. These three directional fluid jets works with the ocular surface anatomy rather than working against it as a standard rinse does.^18^ Although previous studies have demonstrated a reduction in MMP-9 with eye rinsing, ours is the first to illustrate this degree of reduction with a device-facilitated single rinse. The observation that a single rinse can significantly reduce inflammatory expression compared to the standard protocol is meaningful in terms of patient convenience. Multiple attempts to reduce dry eye symptoms can be burdensome, and a device facilitating a superior reduction of MMP-9 via a single rinse suggests a potential advantage in terms of patient ease and adherence to treatment. Moreover, this reduction in MMP-9 continued for some participants up to day 91, which suggests a lasting effect of a singular rinse. It is hypothesized that in these patients with especially long-lasting effects that they most likely had an environmental or external trigger resulting in elevated MMP-9 at baseline. Post rinse, it is thought that this trigger was no longer present in their environment resulting in this prolonged reduction in MMP-9. The study enrollment began in the peak of the spring season 2022 in Ohio, Pennsylvania and West Virginia clinical sites. During the spring season, pollen and allergens impact the overall air quality and can influence presence of MMP-9 in the tear fluid.^19^ This could explain why some patients had elevated MMP-9 during April and May when enrollment began but by the summer months of June and July their allergy season had passed and resulted in this extended duration of reduction in MMP-9. The long-term reduction of MMP-9 expression was not noted for patients enrolled in the standard protocol, as only 1 subject had a negative MMP-9 grading 3 hours post-rinse. Since only one participant in the standard rinse group was negative, it was decided that due to the expense of the MMP-9 POC testing, the standard rinse arm would not be investigated further. Subjectively, for participants in the device arm, DED symptoms improved compared to baseline, as self-reported using the CDES-Q. This improvement in patient symptoms was statistically significant over a standard rinse. The superior reduction in MMP-9 noted in the device arm and the superior improvement in patient symptomatology in the device arm as evidenced by the results of the CDES-Q suggest a correlation that would require further investigation.

Although the results of a single-rinse device for reducing dry eye symptoms and inflammation are suggestive of increased patient accessibility and comfort, this study has some limitations. There was a 28% drop-out rate for day 7 post-rinse and days 30–91. This was primarily because the patients were unable to return to the testing center for multiple visits. This may reflect those patients requiring additional dry eye treatment being inadequately controlled with a single rinse and therefore not returning for follow-up visits, or, conversely, a reflection of those patients who experienced significant and/or sustained improvement, feeling that a follow-up visit was unnecessary. Long-term testing may reveal falsely elevated MMP-9 levels due to the influence of external factors, such as humidity and environmental pollutants. Given that the participants were not masked, there could be an inherent treatment bias to the CDES-Q. The timing of the last post-device rinse of MMP-9 was variable, primarily due to the difficulty of patients returning to the office during the COVID-19 pandemic. Although the study was done during 2022, the timing for a substantial group of participants was during November and December 2022 when there was a resurgence of COVID and flu-like illnesses in the region. This ultimately delayed follow-up visits for rechecks of MMP-9; however, it provided more insight into the durability of the treatment response. Furthermore, the grading of color bands based on intensity using a standardized rubric is a qualitative test of the level of MMP-9, not quantitative. In the future, it may be beneficial to use a quantitative measure of inflammation to obtain more objective results. Finally, by excluding patients who had recent intraocular or refractive surgery, we also acknowledge that we have a subset of patients who require additional investigation as these patients often have significant DED during the post-operative period.

## 4 Conclusions

An irrigating eyelid retractor is a novel medical device that targets fluid at the entire ocular surface to effectively reduce MMP-9 expression 3 hours post-irrigation, with the potential to maintain its effect through 91 days. This approach is superior to standard manual irrigation in its reduction in MMP-9 as well as in its improvement in patients’ symptomatology. This device may serve as an adjunct treatment for DED by offering convenience and tolerability with improved patient symptoms.

## Study approval

The authors confirm that any aspect of the work covered in this manuscript that involved human patients or animals was conducted with the ethical approval of all relevant bodies and the ​study was performed in accordance with the Declaration of Helsinki.

## Funding

This research did not receive any specific grant from funding agencies in the public, commercial, or not-for-profit sectors.

## Declaration of competing interest

The authors declare the following financial interests/personal relationships which may be considered as potential competing interests: SK holds intellectual property for an ittigating eyelid retractor.
